# 不同抗肿瘤治疗方案对肺癌患者新型冠状病毒感染后肺炎发生及严重程度的影响：一项单中心回顾性研究

**DOI:** 10.3779/j.issn.1009-3419.2023.102.20

**Published:** 2023-06-20

**Authors:** Wanjun LU, Jiawen LV, Qin WANG, Yanwen YAO, Dong WANG, Jiayan CHEN, Guannan WU, Xiaoling GU, Huijuan LI, Yajuan CHEN, Hedong HAN, Tangfeng LV, Yong SONG, Ping ZHAN

**Affiliations:** ^1^210002 南京，南京大学附属金陵医院呼吸与危重症医学科; ^1^Department of Respiratory and Critical Care, Jinling Hospital, Affiliated Hospital of Medical School, Nanjing University, Nanjing 210002, China; ^2^210002 南京，东部战区总医院（原南京军区南京总医院）呼吸与危重症医学科; ^2^Department of Respiratory and Critical Care, General Hospital of Eastern Theater Command, People's Liberation Army of China, Nanjing 210002, China; ^3^210002 南京，南京医科大学附属金陵医院呼吸与危重症医学科; ^3^Department of Respiratory and Critical Care, Jinling Hospital, Nanjing Medical University, Nanjing 210002, China

**Keywords:** 肺肿瘤, 新型冠状病毒感染, Lung neoplasms, Corona virus disease 2019

## Abstract

**背景与目的:**

与健康人群相比，肺癌患者新型冠状病毒感染（corona virus disease 2019, COVID-19）的发生率及严重性会增加。目前肺癌的主要治疗方案包括手术、免疫治疗、化疗、放疗、靶向治疗以及抗血管生成治疗，不同抗肿瘤治疗方案对COVID-19的发生及严重性的影响结论尚不统一。本研究旨在探究半年内各种抗肿瘤治疗方案（化疗、靶向治疗、抗血管生成治疗、放疗、免疫治疗和外科手术）是否影响COVID-19后肺炎（以下简称新冠肺炎）的发生率及严重程度。

**方法:**

我们对2022年12月1日-2023年2月15日南京大学附属金陵医院收治的COVID-19且病理确诊为肺恶性肿瘤的217例患者进行了回顾性研究。收集患者临床特征、6个月内抗肿瘤治疗方案以及COVID-19诊断、治疗及转归的数据。通过单因素和多因素Logistic回归分析影响新冠肺炎及影响其严重性的危险因素。

**结果:**

（1）纳入的217例患者中，共51例（23.5%）发生新冠肺炎，其中临床分级为中型42例（82.4%），重型及危重型9例（17.6%）；（2）通过单因素及多因素分析发现超重（OR=2.405, 95%CI: 1.095-5.286）以及肺内病灶放疗（OR=2.977, 95%CI: 1.071-8.274）是影响新冠肺炎发生的危险因素，而全身化疗、靶向治疗以及免疫治疗并不会导致新冠肺炎发生率的增加；（3）在严重程度影响因素的分析中，除了既往有慢性阻塞性肺疾病（chronic obstructive pulmonary disease, COPD）病史（OR=7.600, 95%CI: 1.430-40.387）是重症新冠肺炎的危险因素外，肺内病灶放疗、化疗、靶向治疗以及免疫治疗均不会增加其严重程度。

**结论:**

半年内行肺内病灶放疗导致肺恶性肿瘤患者新冠肺炎的发生率增加，但也并没有增加其严重性，而化疗、靶向治疗、手术和免疫治疗并未导致肺炎的发生及其严重性的增加。

## Abstract (en)

**Background and objective:**

Studies have shown that the incidence and severity of corona virus disease 2019 (COVID-19) in patients with lung cancer are higher than those in healthy people. At present, the main anti-tumor treatments for lung cancer include surgery, immunotherapy, chemotherapy, radiotherapy, targeted therapy and anti-angiogenesis therapy. While the effects of different anti-tumor treatments on the occurrence and severity of COVID-19 pneumonia are not uniform. Therefore, we aimed to describe clinical characteristics and antitumor therapy of patients with lung cancer and COVID-19 pneumonia, and examined risk factors for severity in this population.

**Methods:**

From December 1, 2022 to February 15, 2023, a retrospective study was conducted in 217 patients diagnosed with COVID-19 and pathologically confirmed lung cancer in the Jinling Hospital. We collected data about patients' clinical features, antitumor treatment regimen within 6 months, and the diagnosis and treatment of COVID-19. Risk factors for occurrence and severity of COVID-19 pneumonia were identified by univariable and multivariable Logistic regression models.

**Results:**

(1) Among the 217 patients included, 51 (23.5%) developed COVID-19 pneumonia, of which 42 (82.4%) were classified as medium and 9 (17.6%) were classified as severe; (2) Univariate and multivariate analysis revealed overweight (OR=2.405, 95%CI: 1.095-5.286) and intrapulmonary focal radiotherapy (OR=2.977, 95%CI: 1.071-8.274) are risk factors for increasing occurrence of COVID-19 pneumonia, while other therapies are not; (3) Chronic obstructive pulmonary disease (COPD) history (OR=7.600, 95%CI: 1.430-40.387) was more likely to develop severe pneumonia and anti-tumor therapies such as intrapulmonary focal radiotherapy, chemotherapy, targeted therapy and immunotherapy did not increase severity.

**Conclusion:**

Intrapulmonary focal radiation therapy within 6 months increased the incidence of COVID-19 pneumonia, but did not increase the severity. However, there was no safety concern for chemotherapy, targeted therapy, surgery and immunotherapy.

2019年新型冠状病毒感染（corona virus disease 2019, COVID-19）的爆发使中国乃至全世界的公共卫生遭遇了前所未有的危机，此病毒主要流行特征为传播及变异能力强^[1]^。2020年底，B.1.1.7谱系[于世界卫生组织（World Health Organization, WHO）的变异株命名方案中被称为Alpha]被检测到，并在英国迅速传播。该谱系所包含的几个突变显著改变了病毒表型，包括增强与血管紧张素转换酶2（angiotensin-converting enzyme 2, ACE2）的结合，从而导致了传播性增强。在Alpha谱系出现后不久，另一种病毒谱系B.1.617.2及其后代谱系构成了令人关注的德尔塔（Delta）变异株，并在印度引发了一大波严重急性呼吸综合征冠状病毒2（severe acute respiratory syndrome coronavirus 2, SARS-CoV-2）感染。这一变异株迅速席卷印度，并在世界范围内占据主导地位^[2]^。奥密克戎（Omicron）这种新型变异株，也被称为B.1.1.529，传播迅速，并于2020年11月26日被WHO列为关注的变体（variant of concern, VOC）。进一步的研究^[3]^表明，奥密克戎变异株并不是由早期已知的变异株之一发展而来，这一点从它们基因组之间的几个差异中得到了证明。奥密克戎变异株不是一个单一的菌株，而是演变成多个谱系：BA.1、BA.2和BA.3、BA.4、BA.5^[4]^。2022年12月1日以来的奥密克戎基因组测序提示，BA5.2和BF.7是中国主要流行的变异株类型，占比约80%。感染奥密克戎变异株的患者比感染德尔塔变异株的患者表现出更轻微的症状。此外，有相关研究^[5,6]^表明感染奥密克戎变异株的患者更容易感染上呼吸道而不太可能引起肺部感染。

但对于癌症患者而言，其COVID-19发病率高于普通人群。由于恶性肿瘤状态和抗肿瘤治疗（如化疗或手术）使得患者处于全身免疫抑制状态，因此相比于非癌症患者来说，癌症患者更容易感染。因此，这些患者可能会增加COVID-19发生的风险并且预后较差。同时癌症患者有更高的严重不良事件发生率（需要有创通气或重症监护病房的百分比）^[7⇓-9]^。COVID-19后在某些情况下可诱导过度和异常的无效宿主免疫反应，与潜在致命的严重肺损伤相关，患者可发展为急性呼吸窘迫综合征（acute respiratory distress syndrome, ARDS）^[10]^。

2020年3月一项纳入309例确诊COVID-19的癌症患者的研究^[11]^表明，对于接受化疗的癌症患者，化疗与感染COVID-19的严重程度并没有显著相关性（HR=1.10, 95%CI: 0.73-1.60）。同样地，Lee等^[12]^在2020年4月进行的一项纳入了800例确诊COVID-19的癌症患者的研究中也并没有提供出确切的证据证实与未接受积极治疗的癌症患者相比，接受细胞毒性化疗或其他抗肿瘤治疗的癌症患者因COVID-19导致的死亡风险更高。在单独肺癌的研究数据中同样也表明了COVID-19严重程度的决定因素主要是患者特异性特征，包括吸烟状况和慢性阻塞性肺疾病（chronic obstructive pulmonary disease, COPD），而既往外科手术/放疗和近期全身治疗不影响严重程度^[13]^。但在2020年1月-3月，一项来自湖北武汉的多中心回顾性研究^[14]^纳入205例实验室确认为COVID-19的癌症患者，旨在分析这类人群的基本特征并探究影响死亡率的危险因素，其结果显示癌症患者在出现COVID-19症状的前4周内进行化疗是这类患者在住院期间发生死亡的危险因素。

目前免疫检查点抑制剂（immune checkpoint inhibitors, ICIs）已经成为肺癌患者一线治疗方案，主要包括程序性细胞死亡受体1（programmed cell death 1, PD-1）/程序性细胞死亡配体1（programmed cell death ligand 1, PD-L1）和细胞毒性T淋巴细胞相关蛋白4（cytotoxic T-lymphocyte-associated protein 4, CTLA-4）抗体，主要通过靶向T细胞途径起作用，导致细胞毒性CD8^+^ T细胞的再激活，以增强抗肿瘤活性^[15]^。接受ICIs治疗是患者感染COVID-19严重程度增加的危险因素，这与年龄、癌症类型和其他合并症情况（如慢性肾病、糖尿病等）无关，且研究者在肺癌患者中观察到更严重的COVID-19，但接受ICIs治疗的非肺癌患者也表现出严重的结局^[16]^。支持ICIs暴露可能加剧COVID-19这一观点的一种合理解释与T细胞过度激活机制有关，从而导致呼吸道上皮细胞进一步损伤^[17]^。但Luo等^[18]^对肺癌患者进行的一项研究表明，与从未接受过ICIs的患者相比，无论距离最后一次接受ICIs的时间间隔如何，先前使用过ICIs的患者都不太可能罹患严重的COVID-19。同样地，有研究^[18⇓-20]^表明，PD-（L）1 阻断联合或者不联合化疗并没有增加COVID-19的严重性。Isgro等^[21]^基于ICIs对T细胞活化对抗肿瘤细胞以及病毒感染细胞的积极作用，他们得出结论，在大流行期间，ICIs非癌症患者感染COVID-19的风险因素，并且可能对合并COVID-19的癌症患者具有保护作用。

综上，肺癌患者COVID-19的发生率及严重性均会增加，但抗肿瘤治疗方案对COVID-19后肺炎（以下简称新冠肺炎）的发生及严重性的影响仍存在较大争议，因此本研究旨在探究半年内各种抗肿瘤治疗方案（化疗、靶向治疗、抗血管生长治疗、放疗、免疫治疗和外科手术）是否影响新冠肺炎的发生率及严重程度。

## 1 资料与方法

### 1.1 研究对象

本研究纳入2022年12月1日-2023年2月15日于南京大学附属金陵医院就诊的217例伴有COVID-19的肺癌患者，所有患者经过鼻咽拭子样本定量逆转录聚合酶链反应或者病原体抗原检测确认为COVID-19。COVID-19的诊断基于中国国家卫生健康委员会发布的《新型冠状病毒感染诊疗方案（试行第十版）》；临床分型分为轻型、中型、重型和危重型，其中轻型定义为以上呼吸道感染为主要表现，如咽干、咽痛、咳嗽、发热等，无特征性新冠肺炎表现^[22]^；重症（包括重型及危重型）COVID-19的诊断及危险因素的评估基于中华医学会呼吸病学分会危重症学组撰写的《奥密克戎变异株所致重症新型冠状病毒感染临床救治专家推荐意见》^[23]^。在抗肿瘤治疗过程中，对于排除药物（ICIs）或放疗所致的间质性肺疾病（interstitial lung disease, ILD），需要本科室两名副主任医师级别及以上医生的综合判断。

### 1.2 数据收集

收集COVID-19合并癌症患者的信息，包括患者基本特征、抗肿瘤治疗方案、COVID-19相关记录和胸部计算机断层扫描（computed tomography, CT）图像。其中抗肿瘤治疗方案的分类为：（1）采取以下任何一种抗肿瘤治疗方案（手术、化疗、放疗、靶向治疗、免疫治疗和抗血管生成治疗）均为使用抗肿瘤治疗，而没有行上述治疗的患者为未进行肿瘤治疗；（2）排除手术治疗的患者，根据患者是否采取化疗、放疗、靶向治疗以及免疫治疗进行分类（无患者仅使用抗血管生成治疗）；（3）根据是否联合治疗，分为不同的联合治疗方式，如化疗分为：单独化疗、化疗+抗血管生成治疗、化疗+免疫治疗、化疗+放疗、化疗+靶向治疗、化疗+抗血管生成治疗+免疫治疗、化疗+抗血管生成治疗+放疗、化疗+抗血管生成治疗+靶向治疗、化疗+免疫治疗+放疗、化疗+放疗+靶向治疗及化疗+抗血管生成治疗+免疫治疗+放疗（靶向治疗不推荐联合免疫治疗）等。所有信息均通过定制的数据收集表格获得和整理。两名调查人员负责独立审查数据收集表格，以核实数据的准确性。

### 1.3 统计学处理

采用SPSS 26.0进行统计分析。计量资料以中位数（四分位范围）表示，计数资料采用频数或者百分比表示。采用二元Logistic进行单因素和多因素分析影响新冠肺炎发生的危险因素并分析影响重症新冠肺炎的危险因素；使用卡方检验分析不同放疗方式与新冠肺炎发生的关系。P<0.05为差异有统计学意义。

## 2 结果

### 2.1 患者基本特征

研究纳入的217例COVID-19的肺癌患者基线资料如表1所示，在217例患者中，中位年龄为65岁（34岁-88岁），其中男性153例，女性64例；从未吸烟117例，既往吸烟100例；肺腺癌128例，肺鳞癌42例，小细胞肺癌41例，其他病理类型6例；发生新冠肺炎51例，其中中型42例，重型及危重型9例；无症状感染者19例；最常见的临床症状为发热、咳嗽、乏力、肌肉酸痛、胸闷；胸部CT影像多表现为两肺散在斑片状、片絮状密度增高影，边界不清（图1）。

**表1:** 患者基本临床特征（n=217）

Clinical characteristics		Data
Gender	Male	153 (70.5%)
	Female	64 (29.5%)
Age (yr)	≥65	109 (50.2%)
	<65	108 (49.8%)
History of smoking	Never	117 (53.9%)
	Current or former	100 (46.1%)
COPD	Yes	32 (14.7%)
	No	185 (85.3%)
Hypertension	Yes	85 (39.2%)
	No	132 (60.8%)
Diabetes mellitus	Yes	35 (16.1%)
	No	182 (83.9%)
Cardio-cerebrovascular diseases	Yes	30 (13.8%)
	No	187 (86.2%)
Other tumors	Yes	10 (4.6%)
	No	207 (95.4%)
Overweight	Yes	58 (26.7%)
	No	123 (56.7%)
	Unknow	36 (16.6%)
Type of lung cancer	Adenocarcinoma	128 (59%)
	Squamous	42 (19.4%)
	SCLC	41 (18.9%)
	Others	6 (2.7%)
Distant metastasis	Yes	144 (66.4%)
	No	56 (25.8%)
	unknow	17 (7.8%)
Contralateral lung or pleural metastases	Yes	121 (55.8%)
	No	96 (44.2%)
Gene mutation	Negative	28 (12.9%)
	EGFR	64 (29.5%)
	ALK	5 (2.3%)
	Others	24 (11.1%)
	Unknow	96 (44.2%)
COVID-19 pneumonia	Yes	51 (23.5%)
	No	166 (76.5%)
Severity	Moderate	42 (82.4%)
	Severe	9 (17.6%)

COPD: chronic obstructive pulmonary disease; SCLC: small cell lung cancer; EGFR: epidermal growth factor receptor; ALK: anaplastic lymphoma kinase; COVID-19: corona virus disease 2019.

**图1:**
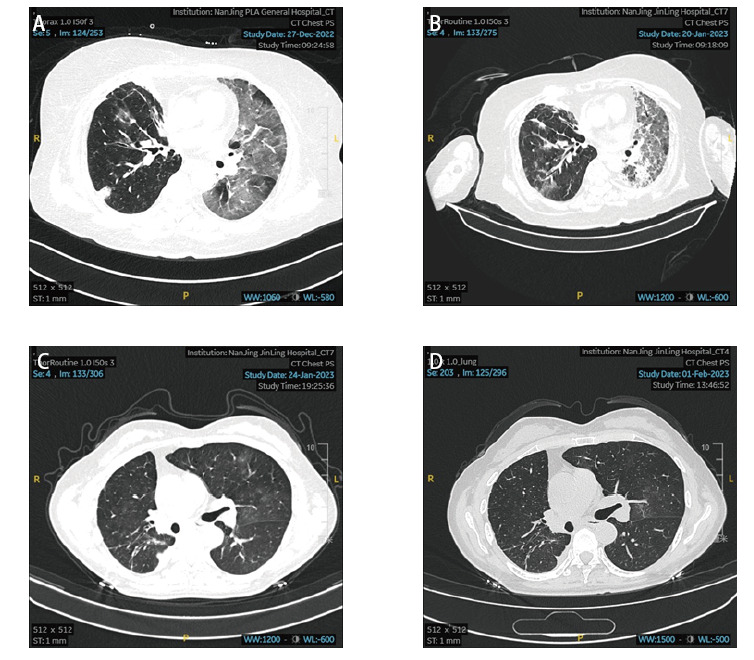
病例1，患者女性，67岁，肺腺癌，半年内行化疗联合免疫治疗以及抗血管生成治疗，危重型肺炎（A：2022年12月26日确诊为新冠肺炎后首次胸部CT影像表现；B：2023年1月20日进展后CT影像学表现）。病例2，患者女性，64岁，肺腺癌，半年内行靶向治疗，中型肺炎（C：2023年1月24日确诊为COVID-19肺炎胸部CT表现；D：2023年2月1日复查胸部CT较前吸收）。

### 2.2 抗肿瘤治疗方案

主要记录2022年12月-2023年2月使用过的抗肿瘤治疗方案，其中未进行任何抗肿瘤方案治疗的患者19例，采用外科手术11例，行肺部病灶放疗20例，化疗116例，靶向治疗56例，免疫治疗99例（表2）。

**表2:** 6个月内抗肿瘤治疗方案

Antitumor treatments		Data
Antitumor therapy	Yes	198 (91.2%)
	No	19 (8.8%)
Surgery	Yes	11 (5.1%)
	No	206 (94.9%)
Intrapulmonary focal radiotherapy*	Yes	20 (9.7%)
	No	186 (90.3%)
Chemotherapy*	Yes	116 (56.3%)
	Chemotherapy	9 (4.4%)
	Chemotherapy+targeted therapy	5 (2.4%)
	Chemotherapy+ICIs	48 (23.3%)
	Chemotherapy+anti-angiogenesis	12 (5.8%)
	Chemotherapy+radiotherapy	5 (2.4%)
	Chemotherapy+targeted therapy+anti-angiogenesis	8 (3.9%)
	Chemotherapy+anti-angiogenesis+ICIs	16 (7.8%)
	Chemotherapy+ICIs+radiotherapy	7 (3.4%)
	Chemotherapy+anti-angiogenesis+radiotherapy	2 (1.0%)
	Chemotherapy+anti-angiogenesis+ICIs+radiotherapy	4 (1.9%)
	No	90 (43.7%)
Targeted therapy*	Yes	56 (27.2%)
	Targeted therapy	33 (16.0%)
	Targeted therapy+chemotherapy	6 (2.9%)
	Targeted therapy+anti-angiogenesis	5 (2.4%)
	Targeted therapy+chemotherapy+anti-angiogenesis	8 (3.9%)
	Targeted therapy+chemotherapy+radiotherapy	2 (1.0%)
	Targeted therapy+radiotherapy	2 (1.0%)
	No	150 (72.8%)
ICIs*	Yes	99 (48.1%)
	ICIs	7 (3.4%)
	ICIs+chemotherapy	48 (23.3%)
	ICIs+anti-angiogenesis	14 (6.8%)
	ICIs+chemotherapy+anti-angiogenesis	16 (7.8%)
	ICIs+radiotherapy	3 (1.5%)
	ICIs+chemotherapy+radiotherapy	7 (3.4%)
	ICIs+chemotherapy+anti-angiogenesis+radiotherapy	4 (1.9%)
	No	107 (51.9%)

ICIs: immune checkpoint inhibitors. *: Adjuvant therapy after surgery cannot be used as a separate treatment, so 11 cases were excluded from surgery within nearly half a year when evaluating the influencing factors of chemotherapy, targeted therapy, radiotherapy and immunotherapy. The total number of cases is 206.

### 2.3 新冠肺炎发生的影响因素

采用二元Logistic单因素及多因素分析探讨影响新冠肺炎发生的危险因素，结果（表3）表明，在单因素分析中超重（OR=2.606, 95%CI: 1.246-5.448）以及半年内行肺部病灶放疗（OR=3.105, 95%CI: 1.202-8.020）是新冠肺炎发生的危险因素，而行外科手术治疗、靶向治疗、化疗及免疫治疗并不会导致新冠肺炎的发生率增加（P>0.05）；将单因素分析结果中P<0.2的影响因素进行多因素统计分析，结果发现超重（OR=2.405, 95%CI: 1.095-5.286）以及肺部病灶放疗（OR=2.977, 95%CI: 1.071-8.274）仍然是影响新冠肺炎发生的危险因素。对于既往行过肺部病灶放疗或者免疫治疗的患者，分析发现放射性肺炎或者免疫相关性肺炎的发生并不会影响新冠肺炎的发生（P=0.418）。对20例行肺部病灶放疗患者进一步分析，将放疗方式进一步分为立体定向放射治疗（stereotactic body radiotherapy, SBRT）和常规分割放射治疗，其中1例患者治疗方式无法明确。分析发现常规分割放射治疗新冠肺炎发生率为63.6%，而SBRT治疗新冠肺炎发生率为25%（表4），但通过卡方检验统计分析并没有显著差异（P=0.115）。

**表3:** 影响新冠肺炎发生因素Logistic分析

Factors	Univariate			Multivariate	
	OR (95%CI)	P		OR (95%CI)	P
Gender (Female vs Male)	0.661 (0.339-1.290)	0.225			
Age (<65 yr vs ≥65 yr)	1.375 (0.724-2.609)	0.330			
History of smoking (No vs Yes)	0.592 (0.307-1.142)	0.118		0.503 (0.232-1.092)	0.082
COPD (No vs Yes)	1.337 (0.571-3.131)	0.503			
Hypertension (No vs Yes)	1.889 (0.991-3.600)	0.053		1.203 (0.530-2.731)	0.658
Diabetes mellitus (No vs Yes)	2.140 (0.985-4.651)	0.055		1.969 (0.75-5.105)	0.164
Cardio-cerebrovascular diseases (No vs Yes)	1.295 (0.532-3.154)	0.569			
Overweight (No vs Yes)	2.606 (1.246-5.448)	0.011		2.405 (1.095-5.286)	0.029
Type of lung cancer (NSCLC vs SCLC)	0.801 (0.499-1.286)	0.359			
Distant metastasis (No vs Yes)	0.992 (0.455-2.163)	0.985			
Contralateral lung or pleural metastases (No vs Yes)	0.828 (0.411-1.668)	0.597			
Gene mutation (No vs Yes)	0.697 (0.267-1.822)	0.462			
Surgery (No vs Yes)	0.31 (0.039-2.498)	0.272			
Antitumor therapy (No vs Yes)	1.790 (0.500-6.418)	0.371			
Intrapulmonary focal radiotherapy (No vs Yes)	3.105 (1.202-8.020)	0.019		2.977 (1.071-8.274)	0.036
Chemotherapy (No vs Yes)	0.983 (0.518-1.869)	0.959			
Chemotherapy combined with others (No vs Yes)	2.700 (0.323-22.587)	0.359			
Targeted therapy (No vs Yes)	1.365 (0.681-2.733)	0.380			
Targeted therapy combined with others (No vs Yes)	1.167 (0.361-3.770)	0.797			
ICIs (No vs Yes)	1.115 (0.589-2.112)	0.738			
ICIs combined with others (No vs Yes)	1.886 (0.215-16.524)	0.567			
Radiation pneumonia/Immune-associated pneumonia (No vs Yes)	0.627 (0.203-1.939)	0.418			

**表4:** 肺部病灶放疗方式与新冠肺炎的发生

Mode of radiotherapy for pulmonary lesions	COVID-19 pneumonia	No COVID-19 pneumonia	Incidence of occurrence
Conventional fractionation radiotherapy	7	4	63.6%
SBRT	2	6	25.0%
Total	9	10	47.4%

SBRT: stereotactic body radiotherapy.

### 2.4 新冠肺炎严重程度影响因素分析

根据《奥密克戎变异株所致重症新型冠状病毒感染临床救治专家推荐意见》，我国近期流行的奥密克戎变异株已有较多重症和死亡病例，因此，我们总结了发生新冠肺炎患者不同临床分型的基本特征（表5），其中在发生新冠肺炎的患者中，重症比例为17.6%。同时我们对影响新冠肺炎严重程度因素进行回归分析，结果发现既往有慢性阻塞性肺疾病是发生重症新冠肺炎的危险因素（OR=7.600, 95%CI: 1.430-40.387）（表6），而半年内行过化疗、放疗、靶向治疗以及免疫治疗并不会增加发生重症新冠肺炎的风险。

**表5:** 不同严重程度新冠肺炎患者的基本特征

Characteristics		Severity	
		Moderate (n=42)	Severe (n=9)
Gender	Male	25 (59.5%)	7 (77.8%)
	Female	17 (40.5%)	2 (22.2%)
Age (yr)	≥65	22 (52.4%)	6 (66.7%)
	<65	20 (47.6%)	3 (33.3%)
History of smoking	Yes	12 (28.6%)	6 (66.7%)
	No	30 (71.4%)	3 (33.3%)
COPD	Yes	5 (11.9%)	4 (44.4%)
	No	37 (88.1%)	5 (55.6%)
Hypertension	Yes	20 (47.6%)	5 (55.6%)
	No	22 (52.4%)	4 (44.4%)
Diabetes mellitus	Yes	10 (23.8%)	2 (22.2%)
	No	32 (76.2%)	7 (77.3%)
Cardio-cerebrovascular diseases	Yes	7 (16.7%)	1 (11.1%)
	No	35 (83.3%)	8 (88.9%)
Overweight	Yes	20 (47.6%)	7 (77.8%)
	No	11 (26.2%)	1 (11.1%)
	Unknown	11 (26.2%)^a^	1 (11.1%)^a^
Type of tumor	SCLC	8 (19.0%)	2 (22.2%)
	Adenocarcinoma	29 (69.1%)	4 (44.4%)
	Squamous	5 (11.9%)	3 (33.3%)
Distant metastasis	Yes	27 (64.3%)	8 (88.9%)
	No	13 (31.0%)	1 (11.1%)
	Unknown	2 (4.8%)^b^	0 (0.0%)
Contralateral lung or pleural metastases	Yes	16 (38.1%)	3 (33.3%)
	No	26 (61.9%)	6 (66.7%)
Gene mutation	Yes	21 (50.0%)	2 (22.2%)
	No	6 (14.3%)	2 (22.2%)
	Unknown	15 (35.7%)^c^	5 (55.6%)^c^
Antitumor therapy	Yes	38 (90.5%)	0 (0.0%)
	No	4 (9.5%)	9 (100.0%)
Surgery	Yes	1 (2.4%)	9 (100.0%)
	No	41 (97.6%)	0 (0.0%)
Intrapulmonary focal radiotherapy	Yes	8 (19.0%)	1 (11.1%)
	No	34 (81.0%)	8 (88.9%)
Chemotherapy	Yes	22 (52.4%)	6 (66.7%)
	No	20 (47.6%)	3 (33.3%)
Chemotherapy combined with others	Yes	22 (52.4%)	5 (55.6%)
	No	0 (0.0%)	1 (11.1%)
Targeted therapy	Yes	15 (35.7%)	1 (11.1%)
	No	27 (64.3%)	8 (88.9%)
Targeted therapy combined with others	Yes	6 (14.3%)	1 (11.1%)
	No	9 (21.4%)	0 (0.0%)
ICIs	Yes	18 (42.9%)	5 (55.6%)
	No	24 (57.1%)	4 (44.4%)
ICIs combined with others	Yes	18 (42.9%)	4 (44.4%)
	No	0 (0.0%)	1 (11.1%)
Radiation pneumonia/immune-associated pneumonia	Yes	3 (7.2%)	1 (11.1%)
	No	39 (92.8%)	8 (88.9%)

^a^: Body mass index (BMI) information was absent in 11 patients with moderate and 1 patient with severe COVID-19 pneumonia; ^b^: 2 patients with moderate COVID-19 pneumonia were missing distant metastasis information; ^c^: loss of gene mutation information in 15 patients with moderate and 5 patients with severe COVID-19 pneumonia.

**表6:** 影响新冠肺炎严重程度危险因素分析

Factors	Univariate analysis	
	OR (95%CI)	P
Gender (Female vs Male)	1.962 (0.354-10.879)	0.441
Age (≥65 yr vs <65 yr)	2.864 (0.519-15.807)	0.227
History of smoking (Yes vs No)	3.846 (0.798-18.531)	0.093
COPD (Yes vs No)	7.600 (1.430-40.387)	0.017
Hypertension (Yes vs No)	1.048 (0.232-4.739)	0.952
Diabetes mellitus (Yes vs No)	1.980 (0.401-9.774)	0.402
Cardio-cerebrovascular diseases (Yes vs No)	0.735 (0.078-6.945)	0.788
BMI	0.963 (0.785-1.182)	0.721
Distant metastasis (Yes vs No)	2.750 (0.302-25.026)	0.369
Intrapulmonary focal radiotherapy (Yes vs No)	1.714 (0.285-10.303)	0.556
Chemotherapy (Yes vs No)	2.727 (0.493-15.095)	0.250
Target therapy (Yes vs No)	0.257 (0.029-2.294)	0.224
ICIs (Yes vs No)	1.211 (0.267-5.489)	0.805
Radiation pneumonia/Immune-associated pneumonia (Yes vs No)	1.905 (0.107-10.481)	0.599

## 3 讨论

在奥密克戎毒株流行之前，肺癌患者相比其他恶性肿瘤患者的感染率更高，且在感染者中肺癌患者的预后更差^[24]^。而抗肿瘤治疗方案，如化疗、手术以及免疫治疗的使用是否会导致COVID-19的发生以及严重程度的增加至今仍未有明确的结论。研究^[5]^表明奥密克戎变异株的感染严重程度低于德尔塔变异株，奥密克戎变异株不能有效地利用跨膜丝氨酸蛋白酶2（recombinant transmembrane protease serine 2, TMPRSS2）进入细胞，但主要依赖于内吞途径，这导致肺实质复制减少，感染上呼吸道的能力增强，使病毒的致病性降低^[3]^。此外，与德尔塔变异株相比，奥密克戎变异株对抗细胞干扰素信号的效果较差，并且核因子κB（nuclear factor kappa B, NF-κB）通路的激活对奥密克戎变异株的反应效率较低^[25]^，这意味着奥密克戎变异株可能诱导较轻的炎症反应^[26]^。而自2022年12月以来，我国新型冠状病毒基因组测序发现奥密克戎变异株BA5.2和BF.7占据全国流行的绝对优势。因此，本研究旨在通过扩大样本量分析2022年12月1日-2023年2月15日确诊为COVID-19的肺癌患者6个月内的抗肿瘤治疗方案是否会导致新冠肺炎的发生及是否增加其严重程度。

我们共纳入217例肺癌合并COVID-19的患者，临床分型基于国家卫生健康委员会发布的《新型冠状病毒感染诊疗方案（试行第十版）》分为轻型、中型、重型。在纳入的患者中，共有51例患者通过胸部CT影像学证实有肺炎的特征性表现而确诊为新冠肺炎。通过单因素及多因素分析发现超重以及肺内病灶放疗是影响新冠肺炎发生的危险因素，而全身化疗、靶向治疗以及免疫治疗并不会导致新冠肺炎发生率增加。在严重程度的影响因素分析中，除了既往有COPD病史的患者是重症新冠肺炎的危险因素外，肺内病灶放疗、化疗、靶向治疗以及免疫治疗均不会增加其严重程度。

在许多恶性肿瘤中，治疗前淋巴细胞减少已被确定为不良预后因素，可能是肿瘤诱导的免疫抑制的反应。此外，已有研究^[27]^表明，治疗后淋巴细胞减少与接受根治性放疗的肺癌患者预后较差有关。因此，在有暴露于感染风险的患者中，放疗诱导的淋巴细胞减少症可能是COVID-19的危险因素^[28]^。但肺内放疗并没有增加COVID-19的严重程度，可能是与化疗相比大多数放疗方案只是中度免疫抑制。因此，Nagar等^[29]^认为在COVID-19大流行期间，放疗可以安全地进行，通常采用低分割方案，这将最大限度地减少前往治疗中心的次数，同时也避免了癌症治疗过程中潜在的有害延误。

关于化疗是否会增加COVID-19严重性，有研究^[12,13]^表明近期的细胞毒性化疗与COVID-19不良结局无关。2020年发表的来自中国武汉的研究数据^[14]^表明在症状出现前4周内的细胞毒性化疗与死亡风险增加相关，接受化疗的患者可能会出现长期的骨髓抑制和免疫功能受损。但该研究更多的血液系统恶性肿瘤患者在症状出现前4周内接受了化疗[血液系统恶性肿瘤20例中有11例（55%） vs 实体瘤162例中有20例（12%）]，这可能部分解释了这些患者预后较差的原因。同样地，关于免疫治疗对于COVID-19的影响也存在较大的争议。Luo等^[18]^发现纳入的患者使用PD-1抑制剂与吸烟状况有关。而在调整吸烟状况后，PD-1抑制剂的暴露与COVID-19严重程度的风险增加无关。PD-1抑制剂不会影响肺癌患者的COVID-19严重程度，且与PD-1抑制剂使用的时间也无显著相关性。一项纳入了11项研究的系统性评价^[30]^发现了中等到高质量的证据，表明ICIs与较高的死亡风险无关；另外有研究^[31,32]^也同样表明ICIs治疗不增加COVID-19的严重程度。但一项纳入110例感染COVID-19且接受了非化疗的ICIs治疗癌症患者的研究^[33]^却表明接受ICIs治疗的癌症患者的住院死亡率很高，并且几乎一半的病例是由于COVID-19。Robilotti等^[16]^对在斯隆-凯特林癌症研究所诊断的423例有症状的COVID-19患者（肺癌患者共35例）进行分析发现接受ICIs治疗是住院和严重疾病的预测因素，而接受化疗和大手术则不是。同样地，对于12个月内接受了免疫治疗的61例癌症患者而言，1/4接受ICIs治疗的COVID-19患者发生死亡，而化疗后的这一比例为13%^[34]^。另外还有研究^[21]^表明免疫治疗是COVID-19的保护因素。我们认为这些结果的异质性来源主要包括大部分研究为回顾性临床研究，纳入的肿瘤类型不尽相同，部分临床研究为小样本研究，且不同的研究中ICIs治疗的时间及治疗方案也不相同。但通过对使用免疫治疗COVID-19患者的转录组学、蛋白组学以及细胞学特征进行分析发现，ICIs治疗并没有改变炎症和I型干扰素反应的诱导^[35]^。而在本研究中，无论是单因素还是多因素分析均没有发现化疗以及免疫治疗会导致新冠肺炎的发生以及严重程度的增加。

本研究主要存在以下局限性：（1）本研究为回顾性临床研究，部分病例通过电话随访，可能存在偏倚；（2）本研究纳入的病例中仅51例发生新冠肺炎，9例为重型及危重型病例，研究结局仅为新冠肺炎的发生以及严重程度分级，未对患者进行长期随访获得更多的生存数据；（3）本研究结果表明肺内病灶放疗导致新冠肺炎的发生风险增加，但因行放疗的病例仅20例患者，病例数较少，结果需要进一步扩大样本证实，另外未进一步根据放疗的剂量范围及分割方式进行分析。

最后，通过研究我们发现半年内抗肿瘤治疗方案中仅肺内病灶放疗导致新冠肺炎的发生风险增加但并没有增加其严重性，化疗、靶向治疗、手术和免疫治疗并未导致肺炎的发生并增加其严重性。在大流行期间不向肿瘤患者提供有效的治疗，确实有增加肿瘤患者死亡率的风险，这可能比COVID-19本身严重得多^[12]^。因此，在疫情流行期间，综合评估患者的基础状况后可考虑继续进行抗肿瘤治疗。

**Competing interests**

The authors declare that they have no competing interests.
