# 更正声明

**DOI:** 10.3779/j.issn.1009-3419.2024.105.01

**Published:** 2024-08-20

本刊2023年第26卷第6期刊登的题名为“不同抗肿瘤治疗方案对肺癌患者新型冠状病毒感染后肺炎发生及严重程度的影响:一项单中心回顾性研究”[陆婉君, 吕嘉文, 王琴, 等. 不同抗肿瘤治疗方案对肺癌患者新型冠状病毒感染后肺炎发生及严重程度的影响:一项单中心回顾性研究. 中国肺癌杂志, 2023, 26(6): 429-438.] 一文中：因作者原因导致单位有误，作者希望修正如下：210002 南京，南京大学医学院附属金陵医院呼吸与危重症医学科（陆婉君，吕嘉文，吕镗烽，宋勇，展平）； 210002 南京，东部战区总医院呼吸与危重症医学科（吕嘉文，王琴，姚艳雯，王栋，吴冠楠，顾晓凌，李慧娟，陈亚娟，韩贺东，吕镗烽，宋勇，展平）；210002 南京，南京医科大学附属金陵医院呼吸与危重症医学科（陈佳妍，吕镗烽，宋勇，展平）。特此更正。作者对此深表歉意。

Erratum: Effect of Different Antitumor Regimens on Incidence and Severity of Corona Virus Disease 2019 Pneumonia in Lung Cancer Patients: A Single-center Retrospective Study

Lu WJ, Lv JW, Wang Q, *et al*.

Zhongguo Feiai Zazhi, 2023, 26(6): 429-438.

In the version of this article initially published, error appeared on page 429 for reason of the authors. The authors' organization should be changed to “210002 南京，南京大学医学院附属金陵医院呼吸与危重症医学科（陆婉君，吕嘉文，吕镗烽，宋勇，展平）； 210002 南京，东部战区总医院呼吸与危重症医学科（吕嘉文，王琴，姚艳雯，王栋，吴冠楠，顾晓凌，李慧娟，陈亚娟，韩贺东，吕镗烽，宋勇，展平）；210002 南京，南京医科大学附属金陵医院呼吸与危重症医学科（陈佳妍，吕镗烽，宋勇，展平）”. The authors expect to make corrections and apologize for the error.

